# Comparison of low‐dose, half‐rotation, cone‐beam CT with electronic portal imaging device for registration of fiducial markers during prostate radiotherapy

**DOI:** 10.1120/jacmp.v14i4.4249

**Published:** 2013-07-08

**Authors:** Ngie Min Ung, Leonard Wee, Sara Lyons Hackett, Andrew Jones, Tee Sin Lim, Christopher Stirling Harper

**Affiliations:** ^1^ School of Physics The University of Western Australia Western Australia Australia; ^2^ Department of Clinical Oncology Faculty of Medicine, University of Malaya Kuala Lumpur Malaysia; ^3^ Elekta Limited Crawley UK; ^4^ Gray Institute for Radiation Oncology and Biology University of Oxford Oxford UK; ^5^ Genesis Cancer Care Wembley Western Australia Australia

**Keywords:** fiducial markers, prostate, IGRT, CBCT, EPID

## Abstract

This study evaluated the agreement of fiducial marker localization between two modalities — an electronic portal imaging device (EPID) and cone‐beam computed tomography (CBCT) — using a low‐dose, half‐rotation scanning protocol. Twenty‐five prostate cancer patients with implanted fiducial markers were enrolled. Before each daily treatment, EPID and half‐rotation CBCT images were acquired. Translational shifts were computed for each modality and two marker‐matching algorithms, seed‐chamfer and grey‐value, were performed for each set of CBCT images. The localization offsets, and systematic and random errors from both modalities were computed. Localization performances for both modalities were compared using Bland‐Altman limits of agreement (LoA) analysis, Deming regression analysis, and Cohen's kappa inter‐rater analysis. The differences in the systematic and random errors between the modalities were within 0.2 mm in all directions. The LoA analysis revealed a 95% agreement limit of the modalities of 2 to 3.5 mm in any given translational direction. Deming regression analysis demonstrated that constant biases existed in the shifts computed by the modalities in the superior–inferior (SI) direction, but no significant proportional biases were identified in any direction. Cohen's kappa analysis showed good agreement between the modalities in prescribing translational corrections of the couch at 3 and 5 mm action levels. Images obtained from EPID and half‐rotation CBCT showed acceptable agreement for registration of fiducial markers. The seed‐chamfer algorithm for tracking of fiducial markers in CBCT datasets yielded better agreement than the grey‐value matching algorithm with EPID‐based registration.

PACS numbers: 87.55.km, 87.55.Qr

## I. INTRODUCTION

Prostate‐implanted fiducial markers have been used as surrogate indicators for the position of the prostate gland for image‐guided radiotherapy (IGRT), and studies have shown that markers are a significantly better surrogate for the true position of the prostate gland than bony landmarks.^1^, ^2^ Fiducial markers have been widely used in conjunction with the electronic portal imaging device (EPID), where stereoscopic rigid‐body registration based on two electronic portal images is adopted as the image registration method for setup verification. However, the EPID images do not provide soft tissue information.

Cone‐beam computed tomography (CBCT) of the patient in the treatment position has gained wider applications for setup verification during radiotherapy. This IGRT method enables therapists to visualize soft‐tissue images of the target organs, as well as the status of surrounding normal structures, during fractionated radiotherapy and therefore provides greater confidence for the use of smaller margins. This is particularly important for target organs, such as the prostate, that are highly mobile and located in close proximity to other critical structures.

Although CBCT provides valuable soft tissue information when the patient is in the treatment position during IGRT, there are three main concerns associated with this imaging modality: the extra imaging dose and time, and the accuracy of localization using various registration (matching) algorithms.

While the imaging dose from the EPID can be easily incorporated into the treatment dose, it is challenging to do the same with CBCT. Several studies have been conducted to investigate the extra dose from imaging received by patients during IGRT using kV‐CBCT. Létourneae et al.^(3)^ reported isocenter and surface dose of 2.8 cGy and 4.4 cGy, respectively, in a 32 cm diameter body phantom from the Elekta XVI kV‐CBCT. Islam et al.^(4)^ reported point doses that ranged from 1.6 to 2.3 cGy per scan at various depths in a cylindrical body phantom using the same CBCT system. Hammoud^(5)^ reported doses range from 1.5 to 2.5 Gy to the skin and 1.3 to 1.8 Gy to the body for 42 fractions of prostate treatments from measurements using thermoluminescent dosimeter (TLD). In a more recent study, Ding et al.^(6)^ reported pelvis scan dose of 1 to 2 cGy for a full rotation pelvis scan. The Ding study also reported that the dose increases up to two times as the patient size decreases. The radiation dose from imaging poses an associated risk and should be minimized.^(7)^ It has been reported that patient position verification by standard mode kV‐CBCT on a daily basis could increase the secondary cancer risk by up to 4%.^(8)^ The use of CBCT is also relatively time‐consuming due to the time taken for scanning and image reconstruction, which may impact treatment sites with large intrafraction setup variation, such as the prostate.

The accuracy of CBCT localization has also been shown to depend on the method and algorithm used respectively to acquire and register the treatment and reference images. Several studies have reported that the use of grey‐value algorithm for matching of soft tissue during prostate radiotherapy does not produce accurate alignment of the prostate in the translational directions during IGRT. Shi et al.^(9)^ compared soft‐tissue–based to fiducial marker‐based manual alignment on 12 prostate patients and found large discrepancies along the superior–inferior (SI) and anterior–posterior (AP) direction alignments, with mean errors of −5.5±4.8mm and −3.1±4.3mm, respectively. Logadóttir et al.^(10)^ compared daily prostate localization for 20 patients on CBCT scans and fiducial markers and concluded that the CBCT soft‐tissue–based setup needs 1 mm larger setup margins than the fiducial‐based setup to account for translational shifts. The Pearson's correlation coefficients of translations in the SI and AP directions for the soft‐tissue–based and fiducial marker‐based matching in the Logadóttir study were 0.24 and 0.48, respectively, indicating modest agreements. Similar results were obtained by Moseley et al.^(11)^ in a study comparing localization performance of fiducial markers and EPID versus soft‐tissue from CBCT images for 15 prostate patients. The results showed relatively weak agreements between the two methods for translations in the SI and AP directions, with Pearson's correlation coefficients of 0.51 and 0.49, respectively.

In the imaging protocols provided by Elekta XVI R4.5 kV‐CBCT system (Elekta Limited, Crawley, UK), volumetric images can be acquired by rotating the kV X‐ray tube and detector through 360° or 200° around the patient. The acquisition time and imaging dose are lower when images are acquired with 200° rotation (hereafter referred to as half‐rotation) rather than 360°. The half‐rotation CBCT protocol is widely used for IGRT of head and neck where there is minimal movement of the target volume and surrounding structures, and is the standard preset for head and neck imaging in Elekta XVI kV‐CBCT. The large number of bony structures at the head and neck enables the image registration procedure to be performed accurately even with the relatively low‐quality images generated from half‐rotation CBCT. This is because the registration algorithm uses the pixel values from the bony structures on the computed tomography (CT) images for matching, and these values are largely unaffected by changing the image acquisition mode from full‐rotation to half‐rotation CBCT since the high contrast of bony structures is preserved.^(12)^ The version of software used in this study also includes an automatic seed‐chamfer matching algorithm for registration of reference and treatment images using fiducial markers as surrogates for the target volume.

In this study, we applied a half‐rotation imaging protocol using kV‐CBCT with localization based on fiducial markers for the correction of setup uncertainties during prostate radiotherapy. The aim is to examine the paired software‐calculated shifts obtained from EPID and half‐rotation kV‐CBCT images using a daily correction strategy for method comparison. The results from this clinical study provide information about the localization performance of half‐rotation CBCT in conjunction with the seed‐chamfer matching algorithm in the clinical environment, compared to the more established method of EPID‐based IGRT of the prostate.

## II. MATERIALS AND METHODS

### A. CBCT dose measurement

Dose measurement was performed based on the method described by Kim et al.^(13)^ to quantify the imaging dose delivered using the half‐rotation CBCT protocol used in this study. In this method, point doses at five different locations in a CT dose index (CTDI) cylindrical body phantom using a CT dose profiler (RTI Electronics AB, Mölndal, Sweden). The point doses measured were considered as the CTDI values, assuming that dose equilibrium was achieved as described in Dixon's point dose method.^(14)^ The weighted CTDI (${\text{CTDI}}_{w}$) which represent the average dose in the phantom, was calculated according to the following formula by Leitz et al:^(15)^

(1) $${\text{CTDI}}_{w}=1/3\;{\text{CTDI}}_{\text{center}}+2/3\;{\text{CTDI}}_{\text{peripheries}}$$

where ${\text{CTDI}}_{\text{center}}$ is a point dose at a central axis and ${\text{CTDI}}_{\text{peripheries}}$ is an average point dose at the peripheries of the body phantom.

Imaging dose of the full‐rotation CBCT were also measured for comparison. The scanning parameters for half‐rotation CBCT and full‐rotation CBCT are shown in Table 1.

**Table 1:** Detailed scan parameters of the half‐rotation and full‐rotation protocols used for imaging dose measurement

	*Scan Protocol*	
	*Half‐rotation CBCT*	*Full‐rotation CBCT*
Peak voltage (kVp)	120	120
Number of frames	366	660
Nominal mAs per frame	16	16
Nominal ms per frame	20	20
Total mAs	117.1	211.2
Acquisition angle (deg)	200	360
Gantry rotation speed (deg/min)	180	180
Scanning time (sec)	67	120

### B. Patient cohort

A group of 25 consented prostate cancer patients with implanted fiducial markers were treated with radical intent by three‐dimensional conformal radiation therapy or intensity‐modulated radiotherapy using Elekta Synergy or Elekta Axess linear accelerators (Elekta Limited). Each patient was implanted with Acculoc 99% pure gold fiducial markers (Civco Medical Systems, IA, USA) measuring 3 mm length by 1.2 mm diameter, not less than one week prior to planning CT. Each patient was also briefed about drinking 375 ml of water after voiding approximately 1 hour prior to treatment. Out of the 25 patients, 21 were prescribed with 74 Gy in 37 daily fractions, while the remaining four were prescribed with 76 Gy in 38 fractions.

### C. Image acquisition

To compare the localization performance using half‐rotation CBCT and EPID, images were acquired with both modalities before each daily treatment session. Portal images were taken immediately after setup of the patient on the couch based on the position of lasers and skin tattoos. Portal images were acquired at subtended angles between 50° to 130° and stored for off‐line analysis.

A CBCT dataset with slice thickness of 2 mm was acquired using the half‐rotation CBCT protocol with X‐ray tube setting of 120 kVp and 117 mAs immediately after the acquisition of portal images and before treatment. The reconstruction resolution was set at 1 mm^3^ and a filtered back‐projection technique was employed in the reconstruction of 3D volumes.^(16)^

Seed‐chamfer registration was performed to match the CBCT scans with the planning CT using the XVI software version 4.5 (Elekta Limited). The isocenter shifts in three translational and rotational axes were computed. However, only the translational couch correction was performed, as the patient support system was unable to correct for the rotational components at the time in this study.

### D. Image registration

An off‐line stereoscopic image registration was performed on the portal image pairs using MOSAIQ R1.6 (Elekta/Impac Medical Systems, Sunnyvale CA). Both images were scaled manually before the image registration process. The isocenters of the portal images were determined using a process called field edge detection. In this process, the edges of the portal field, which were defined by the multileaf collimators, were first identified. These field edges were transposed onto the corresponding DRRs and adjusted, if necessary, to obtain the best fit within the DRR field. Once this was done, the position of the isocenter was established in the portal images. The fiducial markers visible on both treatment portals were then identified and matched to the reference DRRs using the point‐based rigid body registration algorithm. The translations in the three cardinal axes were recorded.

Two different methods were used to register the volumetric CBCT images with the reference CT images in this comparison study. The first registration method was the seed‐chamfer technique, which was used for online correction during treatment. In this method, the ‘mask’ tool was used to mark all the fiducial markers visible on the CBCT scans. The seed‐chamfer algorithm selects only high‐density pixels with at least 1.5 times the density of water, presumed to correspond to fiducial markers, for registration. The XVI software computed the deviations of the positions of the fiducial markers between the localization CBCT images and reference CT images, and the results were used for online correction.

The second image registration method, which was performed off‐line, was the grey‐value matching technique. In this technique, a ‘clipbox’ was used to define the region of interest for grey‐value registration. This clipbox encompassed the PTV and all fiducial markers identified in the CBCT images. The grey‐value algorithm uses all the pixel information, including that of the fiducial markers and the surrounding soft tissue inside the clipbox, for matching using the grey‐level correlation ratio algorithm.^(17)^

Both CBCT registration algorithms/methods use information about the fiducial markers for registration. However, the algorithms assign different importance or priority to the various surrogates for registration; the seed‐chamfer and grey‐value algorithms designate the fiducial markers and soft tissue, respectively, as priority surrogates for registration.

The XVI software can compute the registration results in terms of translations and rotations about three axes. However, for the purpose of this study, the rotational component of the XVI software was disabled to allow computation in the translational components only. This is because the version of MOSAIQ used during the study for performing stereoscopic registration of portal images was unable to solve for rotational components. Off‐line analysis was performed by a single expert user to eliminate interobserver variability. Online analysis was performed by various users during treatment but the interobserver variability was anticipated to be minimal, as matching of fiducial markers was automated by the XVI software using the seed‐chamfer algorithm.

### E. Statistical analysis

Analysis was performed for the translational shifts of the isocenter position along the three axes, AP, LR and SI, for each daily session for each patient. The mean systematic error (M), systematic error (X) and random error (a) were calculated for each registration method from the software‐suggested shifts based on the method described by van Herk.^(18)^

Agreement between the two registration methods was assessed using the 95% limits of agreement (LoA) analysis with multiple observations per patient.^(19)^ The LoA method allows the user to assess the agreement between datasets obtained from two methods from a plot of the differences (y‐axis) versus the mean of the two measurements (x‐axis). The plots also include two reference lines, representing the 95% LoA, which were estimated as the mean difference ±1.96 multiplied by the standard deviation of the difference.

Deming orthogonal regression was performed to evaluate the bias‐corrected correlation between paired measurements using the ratio of variances between EPID and CBCT cohorts, as well as the proportional and constant biases between methods.^(20)^ The clinical policy in our institution requires couch correction to be executed if there is a shift of 5 mm or greater in any translational direction for verification using EPID. Cohen's kappa inter‐rater analysis^(21)^ was performed to compare the agreement between the two image‐guidance methods for prescribing couch corrections at 3 and 5 mm action levels. The aim of this analysis was to evaluate the agreement between the two methods for selecting one of two possible actions: ‘perform couch correction’ and ‘do not perform couch correction'. The Cohen's kappa coefficient κ indicates the proportion of agreements that was observed between the image‐guidance methods, after adjusting for the proportion of agreements that occurred by chance. The computed values of κ range from 0 to 1, with the value of 1 indicating perfect (complete agreement). Landis and Koch^(22)^ proposed a guideline to interpret the values of κ into the ordinal scale, whereby comparisons yielding values κ larger than 0.81 are classed as ‘very good', larger than 0.61 as ‘good', larger than 0.41 as ‘moderate', larger than 0.21 as fair, and no larger than 0.20 as ‘poor'.

## III. RESULTS

A total of 873 paired software‐suggested shifts of half‐rotation CBCT and EPID were captured during the course of radiotherapy for the 25 patients in this study, with a mean of 37 alignment pairs per patient.

Dose measurement using a CTDI body phantom revealed that the half‐rotation CBCT protocol used in this study delivers a ${\text{CTDI}}_{w}$ of 3.7 mGy per scan, compared to 6.7 mGy from the full‐rotation CBCT protocol.

The values of M,Σ, and σ for both modalities are shown in Table 2. The differences in the values of M,Σ, and σ between registrations based on EPID and half‐rotation CBCT using seed‐chamfer and grey‐value matching algorithms were within 0.2 mm in all directions. They were also observed to be the largest in the AP direction. The random errors (σ) were consistently larger than the systematic errors (Σ) by approximately 1 mm in all directions.

The method comparison using the LoA analysis is shown in Table 3. The comparison of registrations with EPID and half‐rotation CBCT (seed‐chamfer) resulted in mean differences of 0.2±1.2mm,−0.4±1.5mm, and 0.0±1.4mm in the LR, SI, and AP directions, respectively. The 95% LoA were −2.5 to 2.5 mm, −3.4 to 2.4 mm, and −2.8 to 2.8 mm in the LR, SI, and AP directions, respectively. The comparison using EPID and half‐rotation CBCT (grey‐value) showed relatively poor agreement, with mean differences of 0.1±1.3mm,−0.6±1.7mm, and 0.2±1.6mm in the LR, SI, and AP directions, respectively, and 95% LoA of −2.5 to 2.8 mm, −3.8 to 2.7 mm, and −3.0 to 3.4 mm in the LR, SI, and AP directions, respectively. Both comparisons revealed the narrowest and widest 95% LoA in the LR and SI directions, respectively.

**Figure 1:**
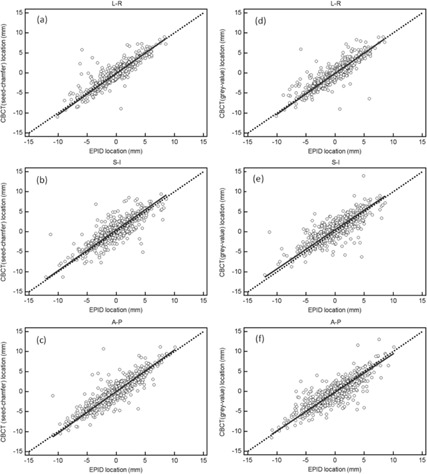
Scatter plots of translational shifts from half‐rotation CBCT (seed‐chamfer) and EPID in the (a) LR, (b) SI, and (c) AP directions, as well as the translational shifts from half‐rotation CBCT (grey‐value) and EPID in the (d) LR, (e) SI, and (f) AP directions. The solid line is the Deming regression fit and the dotted line is the line of identity.

Figure 1 shows the bivariate scatter plots, with Deming regression fit and line of identity, comparing EPID and half‐rotation CBCT for localization. The solid line represents the Deming regression fit and the dashed line represents the line of identity. If both imaging modalities agreed perfectly, the Deming regression fit would coincide exactly with the line of identity. Visually, the comparison between results of registrations with EPID and half‐rotation CBCT (seed‐chamfer) show near‐perfect agreement, with the Deming regression fit almost identical to the line of identity. The proportional and constant biases between methods are summarized in Table 3. Comparisons generally showed good agreement, with no constant or proportional bias at p<0.05. However, nonzero constant biases were exhibited in the SI direction in the comparisons of registrations with EPID versus half‐rotation CBCT for both seed‐chamfer and grey‐value matching algorithms.

Table 4 summarizes the results of Cohen's kappa analysis comparing the agreement between methods/matching techniques in prescribing translational corrections of the couch at 5 mm action level. The κ values obtained were 0.65, 0.61, and 0.68 in the LR, SI, and AP directions, respectively, for the comparison between EPID and half‐rotation CBCT using seed‐chamfer algorithm, which correspond to ‘good’ agreements according to the Landis and Koch criteria. The comparison between registrations with EPID and half‐rotation CBCT using grey‐value matching yielded slightly lower κ values of 0.61, 0.59, and 0.60 in the LR, SI, and AP directions, respectively. Both comparisons yielded the best agreement in the LR direction. Table 5 summarizes the results of Cohen's kappa analysis comparing agreement between

**Table 2:** The translational errors M,Σ, and σ in the LR, SI, and AP directions for image registration with EPID and half‐rotation CBCT (seed‐chamfer and grey‐value matching algorithms) (all values in mm)

*Modality/Registration Technique*	*LR*			*SI*			*AP*		
	*M*	Σ	σ	*M*	Σ	σ	*M*	Σ	σ
EPID	−0.1	1.4	2.3	−0.2	1.5	2.5	−0.3	1.7	2.7
CBCT (seed‐chamfer)	−0.3	1.4	2.4	0.3	1.4	2.6	−0.3	1.7	2.8
CBCT (grey‐value)	−0.2	1.3	2.4	0.4	1.4	2.5	−0.5	1.5	2.7

**Table 3:** The results of Bland‐Altman LoA analysis for method comparison between EPID and half‐rotation CBCT (mm)

	*LR*		*SI*		*AP*	
*Modality/Registration Technique*	*Difference* (Mean±SD)	*95% LoA*	*Difference* (Mean±SD)	*95% LoA*	*Difference* (Mean±SD)	*95% LoA*
EPID vs. CBCT (seed‐chamfer)	0.2±1.2	−2.5 to 2.5	−0.4±1.5	−3.4 to 2.4	0.0±1.4	−2.8 to 2.8
EPID vs. CBCT (grey‐value)	0.1±1.3	−2.5 to 2.8	−0.6±1.7	−3.8 to 2.7	0.2±1.6	−3.0 to 3.4

**Table 4:** The Cohen's kappa coefficient (κ) for method comparison in executing translational couch correction at 5 mm action level. The agreement in ordinal scale is according to the Landis & Koch criteria.^(22)^

*Modality/Registration Technique*	*LR*		*SI*		*AP*	
	κ(95%CI)	*Agreement*	κ(95%CI)	*Agreement*	κ(95%CI)	*Agreement*
EPID vs. CBCT (seed‐chamfer)	0.65 (0.61 to 0.69)	Good	0.61 (0.57 to 0.65)	Good	0.68 (0.65 to 0.72)	Good
EPID vs. CBCT (grey‐value)	0.61 (0.57 to 0.65)	Good	0.59 (0.55 to 0.63)	Moderate	0.60 (0.55 to 0.63)	Moderate

Methods/matching techniques in executing translational corrections of the couch at a 3 mm action level. The results were similar to those for an action level of 5 mm. Kappa coefficients were generally higher for the 3 mm action level in the LR and AP directions compared to the 5 mm action level. Intermodality comparison between EPID and CBCT (seed‐chamfer and grey‐value algorithms) yielded moderate and good agreement with kappa coefficients ranging from 0.53 to 0.75 in all directions. Intramodality comparison between seed‐chamfer and bony‐match algorithms showed poor agreement in the SI and AP directions with kappa coefficients of 0.18 and 0.28, respectively.

**Table 5:** The Cohen's kappa coefficient (κ) for method comparison in executing translational couch correction at 3 mm action level. The agreement in ordinal scale is according to the Landis & Koch criteria.^(22)^

*Modality/Registration Technique*	*LR*		*SI*		*AP*	
	κ(95%CI)	*Agreement*	κ(95%CI)	*Agreement*	κ(95%CI)	*Agreement*
EPID vs. CBCT (seed‐chamfer)	0.75 (0.72 to 0.79)	Good	0.60 (0.56 to 0.63)	Moderate	0.71 (0.68 to 0.74)	Good
EPID vs. CBCT (grey‐value)	0.71 (0.68 to 0.75)	Good	0.53 (0.49 to 0.57)	Moderate	0.63 (0.59 to 0.66)	Good

## IV. DISCUSSION

This study has demonstrated that CBCT‐based registration of fiducial markers can be considered consistent with the more established technique of EPID‐based registration, particularly if the seed‐chamfer algorithm is used to register the CBCT dataset. Further, the images obtained from the half‐rotation protocol are of sufficient quality for registration of fiducial markers. The Bland‐Altman LoA analysis showed that 95% of registrations performed with CBCT in conjunction with the seed‐chamfer algorithm would yield translations that would agree with EPID‐based registrations to within 2.0 to 3.5 mm. The agreement limits are comparable with the results of the investigation by Moseley et al.^(11)^ and Owen et al.^(23)^ with the limits of 1.5 to 4.0 mm and 0.2 to 3.9 mm, respectively. (Note that the LoA of the Owen study were set to a 3 mm threshold.) In all studies, the LoA are poorest in the SI direction. The LoA method does not prescribe a threshold for agreement, as this should be set according to clinical requirement. In the case of this study, the LoA of the translational shifts were all within the nonzero action level for of 5 mm for couch correction. Therefore, it is concluded that the two methods of localization can be used interchangeably at a 5 mm action level.

Deming regression analysis identified constant biases between registrations with EPID and half‐rotation CBCT in the translational SI direction. This was probably due to the finite thickness of CT slices (2 mm) used along the SI direction, which limited the accuracy of registration in this axis. No proportional bias was observed for any method comparison. It should be noted that a constant bias is easy to account for using a linear differential, as opposed to the proportional error component which may exhibit a different mathematical relationship.

The results of the Cohen's kappa inter‐rater analysis were consistent with the LoA analysis, and confirmed that the two image‐guidance methods can be considered interchangeable at a 5 mm action level. A detailed assessment of the coefficients revealed the best and worst agreement in the LR and SI directions, respectively, which is consistent with other method comparison studies.^10^, ^11^, ^23^

The seed‐chamfer matching algorithm of half‐rotation CBCT has been shown to consistently yield better agreement with EPID‐based registration than the grey‐value matching algorithm for translational shifts. The seed‐chamfer algorithm uses only pixels with density at least 1.5 times that of water, presumed to correspond only to fiducial markers, whereas the grey‐value algorithm uses all pixels within the clipbox for registration. It has been reported that the rigid‐body registration of soft tissue alone for translational shift is not as accurate in the presence of organ deformation due to temporal physiologic activities^24^, ^25^, ^26^ and this may explain the slight inferiority of the grey‐value algorithm compared to the seed‐chamfer algorithm in achieving agreement with the fiducial‐based registration with EPID.

The results obtained from this study were purely from the software itself with minimal human intervention, as the aim of this study is to examine the paired software‐calculated shifts obtained from EPID and half‐rotation kV‐CBCT images. The only intervention performed was in the adjustment of the clipbox for registration for any invalid image registration. After obtaining the registration results, the treatment staffs carried out manual intervention (for example, manually corrected for the registration for any substandard software registration). However, the results of any significant manual adjustment were not considered in this study.

A few factors may contribute to the discrepancies between half‐rotation CBCT and EPID for localization. Firstly, the finite thickness of CBCT slices limited the accuracy for determining position of fiducial markers in the SI direction. Secondly, the relatively poor quality of EPID images obtained using EPID may reduce the accuracy of identifying fiducial markers. Thirdly, there was a time gap (approximately 2 minutes) between the acquisition of the CBCT scan and EPID images, and accuracy of localization was thus subjected to the intrafraction displacement of the prostate. Some of the factors were larger on certain treatment fractions, thus causing some outliers in the comparison dataset, as can be observed from Fig. 1. For example, in some treatment fraction, the very poor image quality obtained using EPID caused large uncertainty in identifying the markers. However, it is expected that these outliers do not contribute significantly to the mean and root mean square difference between the two methods, since the number of these outliers is relatively small.

The presence fiducial markers contributed significantly to the matching in the grey‐value matching algorithm where the success rate was very high. This result is in contrast to the one described by Smitsman et al.^(17)^ as they did not use fiducial markers in their grey‐value matching. There were some instances where the matching of fiducial markers failed due to significant rotation, but that occurred in very small number.

Our results have shown that we can use half‐rotation CBCT and EPID as image‐guidance methods interchangeably. In situations where we use half‐rotation CBCT for image guidance specifically for prostate patients implanted with fiducial markers, we have EPID as a backup modality in the case of equipment failure in imaging on any treatment day, or vice versa. This study has also demonstrated the feasibility of using half‐rotation CBCT protocol for daily setup correction during the IGRT of prostate. The additional information of soft tissues from the CBCT scans enables the treatment staff to detect substantial changes in the status of the rectum and bladder during radiotherapy of the prostate. During this clinical study, a few patients have been counseled on the importance of strictly observing the bowel preparation protocol because of consistent variability of the volume and/or position of the rectum and bladder during radiotherapy.

The half‐rotation CBCT protocol used in this study reduced the imaging dose by reducing the scan angle by approximately half and subsequently decreasing the number of acquired frames from 660 to 366. If pretreatment scans are recorded before each fraction, the total imaging dose delivered using the half‐rotation CBCT protocol would be around 14 cGy, which is approximately 0.2% of the total prescribed treatment dose of 74 Gy or 76 Gy. Adopting 0.2% of a treatment dose as the imaging dose limit recommended by Murphy et al.,^(27)^ the half‐rotation CBCT protocol used in this study would satisfy this dose limit. Further, we have found in a separate study that the of image quality obtained from the half‐rotation protocol was comparable to that obtained from a full rotation. This is consistent with the findings by Kowatsch et al.,^(28)^ where a reduction in projection data due to reduced scan angle did not significantly deteriorate the image quality parameters.

A limitation of this prospective study is that the rotational displacement cannot be determined from the version of MOSAIQ software used for stereoscopic registration using EPID. A study by Logadóttir et al.^(10)^ reported that soft‐tissue–based verification is more accurate than fiducial marker‐based verification for determining the rotational shift. Also, as per our institutional protocol, the XVI software calculated the shifts using six degrees of freedom, which included rotational shifts around three axes. We recognized that a large rotation may affect the shift in the translational directional, but this will not impact significantly the results of our comparison study here as the rotational errors in our cohort of patient are generally small (mean of 0.7°).

Another limitation is that the fiducial marker migration was not considered in this study. However, studies have shown that fiducial marker migration during a course of radiotherapy is minimal.^29^, ^30^ Furthermore, fiducial marker migration is anticipated to have little effect on the statistical analysis for method comparison, as any change in the position of fiducial markers should affect all imaging modalities. The fiducial marker migration is also expected to have less impact on the grey‐value registration than on the seed‐chamber registration.

## V. CONCLUSIONS

There is acceptable agreement between registrations of fiducial markers with EPID and half‐rotation CBCT. Registrations of the CBCT datasets using the seed‐chamfer algorithm resulted in maximum 95% LoA of 3.5 mm with stereoscopic registration of portal images. The maximum 95% LoA between grey‐value registrations of CBCT datasets and portal images was 3.8 mm. The half‐rotation CBCT‐ and EPID‐derived translational shifts were shown to be equivalent for couch correction at 3 and 5 mm action level and thus can be used interchangeably. The setup uncertainties of the imaging modalities are similar in magnitude, with the largest uncertainty in the AP direction. This information can be used to design the image‐guidance strategy and treatment margins for radiotherapy of the prostate.

This study has shown that low‐dose, half‐rotation CBCT is feasible for daily online image guidance of the prostate implanted with fiducial markers. The accuracy of this method is comparable to that of EPID and also provides images of acceptable image quality for the visualization of targeted critical organs, as well as for the assessment of treatment response.

## ACKNOWLEDGMENTS

The authors would like to thank Simon Woodings and all radiation therapists at Genesis Cancer Care WA who were involved in this study. The corresponding author also acknowledges financial support from the Ministry of Higher Education in Malaysia and the University of Malaya.
